# Glycan-specific whole cell affinity chromatography: A versatile microbial adhesion platform

**DOI:** 10.1016/j.mex.2014.10.005

**Published:** 2014-10-22

**Authors:** Maxwell L. Van Tassell, Neil P.J. Price, Michael J. Miller

**Affiliations:** a Department of Food Science and Human Nutrition, University of Illinois, Urbana, IL 61801, USA; b National Center for Agricultural Utilization Research, Agricultural Research Service, U.S. Department of Agriculture, Peoria, IL 61604, USA

**Keywords:** Affinity chromatography, Bacterial adhesion, Carbohydrate modification, Screening, Lectins, *Escherichia coli*, *Campylobacter jejuni*

## Abstract

We have sought a universal platform for elucidating and exploiting specificity of glycan-mediated adhesion by potentially uncharacterized microorganisms. Several techniques exist to explore microbial interactions with carbohydrate structures. Many are unsuitable for investigating specific mechanisms or uncharacterized organisms, requiring pure cultures, labeling techniques, expensive equipment, or other limitations such as questionable stability, stereospecificity, or scalability. We have adapted an affinity chromatography resin as a model to overcome these drawbacks, among others. It readily allows for the quantification, selection, and manipulation of target organisms based on interactions with glycan ligands. To maximize its utility as a selective screening method, we have constructed the tool such that it:

• Promotes whole-cell interactions using viable, unaltered cells.
• Provides robust spatial interactions with target glycans, presented with controlled stereo-specificity, for high affinity/avidity interactions that reflect a complex *in vivo* matrix.
• Has the ability to utilize any reducing glycan, is quick, efficient, safe, and affordable to construct, and is scalable and reusable for multiple applications.

## Graphical abstract

**Figure:**
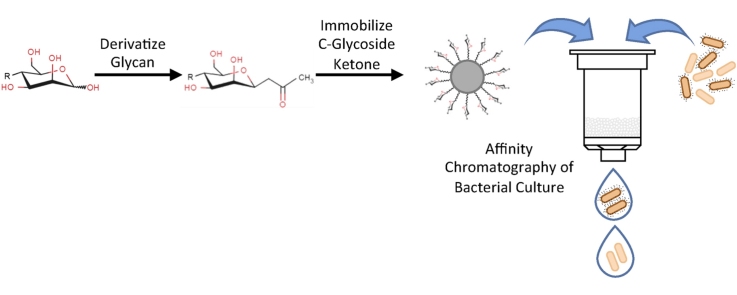

## Method details

### Part 1: Formation of *C*-glycoside ketones and their immobilization

In brief, the affinity chromatography model construction consists of derivatizing a target glycan with a ketone modification at the reducing end to facilitate covalent attachment to a hydrazide-functionalized resin. Quantitative conversion of a glycan to its *C*-glycoside ketone derivative can be accomplished as described previously [1] via a straightforward, one-pot reaction requiring no activating or protecting groups. Ketone derivatization makes the glycoside highly reactive toward hydrazines, facilitating rapid immobilization onto hydrazide-modified chromatographic resin. Furthermore, formation of a *C*-glycoside ketone derivative ensures a stereospecific modification that maintains the ring integrity of the glycoside, which may be essential for proper presentation to cell adhesins. For more information on the derivatization chemistry, see [2].

The resulting *C*-glycoside ketohydrazide resin could be constructed with any carbohydrate with an aldose residue at its reducing end. The reactions detailed below are scalable as long as the molar ratio of the components is equivalent; we have successfully derivatized over 5 mmol of carbohydrate and made batches of resin up to 5 mL. While we have found that the specified hydrazide-functionalized resin works well, any other hydrazide-functional substrate should also work, but may require an altered ratio of *C*-glycoside ketone.

#### Materials

• Glycan(s) of interest
• Sodium bicarbonate buffer: 38 g L^−1^ NaHCO_3_, pH 8.5
• Acetyl acetone
• Ethyl acetate
• Dowex 50WX8 strong acid ion exchange resin (Sigma–Aldrich, cat. 217492)
• Ultralink hydrazide resin (Thermo Scientific, cat. 53149)
• Pyridine
• Sodium azide

*Note*: Materials lists include only non-standard items. Common laboratory ware and equipment such as water baths are assumed to be available.

#### Procedure

A1 In a glass screw-cap tube, dissolve 0.3 mmol of a desired glycan in 1 mL of sodium bicarbonate buffer.
A2 Add 35 μL of acetyl acetone (approx. 1.1:1 molar ratio with glycan) and vortex to dissolve.
A3 Incubate sealed glass tube in a water bath or heating block at 80–90 °C for at least 4 h.
A4 Remove tube and cool to ambient temperature. A slight yellow-brown discoloration may develop, particularly with larger or more complex glycans.
  a. A small amount of pressure from CO_2_ release may build within the tube during incubation. Vent the tube slowly prior to opening and it should not be problematic.
A5 Add 3 mL of ethyl acetate and shake to mix. Allow ethyl acetate to separate from the aqueous phase and discard this upper layer.
  a. If the ethyl acetate is slow to separate, place the reaction in a freezer for 10–15 min to help.
A6 Add acid ion exchange resin to the aqueous phase, 10–20 mg at a time in order to neutralize. Shake gently to release CO_2_ and add more resin until no more gas is generated.
A7 Remove the aqueous phase, containing the newly formed *C*-glycoside ketone, from the ion exchange resin and retain.
  a. *C*-glycoside ketone formation may be verified by mass spectrometry at this stage if desired.
A8 Prepare 1 mL (bed volume) of hydrazide resin in a glass vial and wash with water to remove storage buffer.
A9 Remove excess water from the resin and apply 200 μL of the *C*-glycoside ketone solution.
A10 Evaporate the majority of the aqueous phase from the resin under an air stream, carefully so as to not displace the resin from the vial.
A11 Dilute the resin in 2 mL pyridine, vortex to mix, and incubate at ambient temperature for at least 1 h.
A12 Carefully remove pyridine with a Pasteur pipette and wash the resin in 3–4 volumes of water. Wash 5–6 times to remove all pyridine.
A13 Retain the resin for use or suspend in 0.02% sodium azide for 4 °C storage. We have found that the resin remains functional for longer than two months.

### Part 2: *C*-Glycoside resin verification

**Fig. 1:**
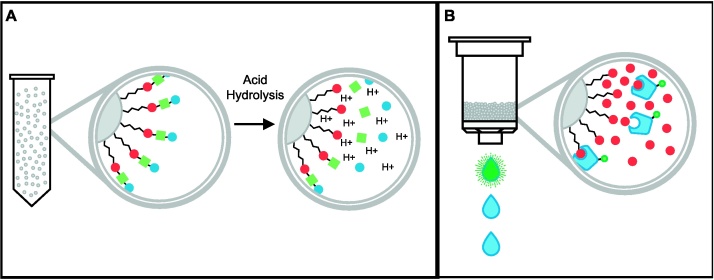
Example methods for analysis of *C*-glycoside ketone immobilization. (A) Acid hydrolysis of the glycosidic bonds within the immobilized glycan releases component monomers, except for the ketone-modified terminal residue bound to the hydrazide resin. Immobilization of the *C*-glycoside ketone could be verified by examining the hydrolysate via various analytical means, including HPLC, enzymatic assay, or mass-spectrometry. (B) Attachment of a lectin or set of lectins of known specificity compatible with the target glycan can also verify its immobilization. Elution of the lectin via ligand competition demonstrates that the lectin was adherent in a glycan-specific fashion, indicating the glycan was immobilized with a structurally compatible presentation.

Once the above procedure has been completed, validation that the target glycan has been immobilized may be desired. We have found that if the glycan is a disaccharide or larger, glycosidic linkages can be acid-hydrolyzed to release constituent monomers for analysis, by mass-spectrometry for example, as described below. The ketone-modified monosaccharide from the reducing terminus of the glycan, which has been immobilized to the hydrazide resin, is not released by acid hydrolysis (Fig. 1A); it should be the sole component not detected by further analysis such as mass-spectrometry (see [3,4] for more on analytical methods).

Alternatively, a lectin-affinity assay can be performed to confirm immobilization of glycans. This is particularly useful for immobilized monosaccharides, which are not readily acid-hydrolysable. It can also verify structural conformation of the carbohydrate presented on the resin, however it may require optimization for each application. Lectins with appropriate specificities for target glycan components must be used. Similarly, the lectin load may vary depending on label and a compatible buffer, as suggested by the supplier, must be used to ensure effective adhesion of the lectin. For example, we have used Concanavalin A to validate the successful immobilization of alpha-d-mannose (see Fig. 1B).

#### Materials

• *C*-glycoside ketohydrazide resin, as prepared above in Part 1
• 2 N Trifluoroacetic acid (TFA)
• Hydroxylamine HCl, saturated solution in pyridine
• Acetic anhydride
• Ethyl acetate for extractions
• Stoppered spin columns with 30 μm pore filters (Thermo Scientific, cat. 69725)
• Fluorescently labeled lectin(s) specific for target glycan
o e.g. Fluorescein labeled Concanavalin A (Vector Labs)
• Lectin buffer compatible with desired lectin (see suppliers’ recommendations)
o e.g. 10 mM HEPES, 0.15 M NaCl, 0.1 mM CaCl_2_, pH 7.5
• Fluorescence microplate reader and compatible 96-well fluorescence microplates

#### Procedure – hydrolysis and mass spectrometry

A1 150 μL of resin was placed in a glass vial with 0.5 mL of 2 N TFA and hydrolyzed on a heating block at 120 °C for 30 min. This was then evaporated to dryness on an airline.
A2 0.5 mL saturated hydroxylamine HCl in pyridine was added to the dried residue and incubated for another 20 min at 60 °C.
A3 0.5 mL acetic anhydride was added and reacted for another 20 min at 60 °C, to form aldononitrile acetate derivatives. The reaction was evaporated (fume hood), re-dissolved in ethyl acetate, and back extracted with water. The upper, ethyl acetate layer was removed and analyzed by GC/MS as described [5].

#### Procedure – affinity chromatography with lectins

B1 100 μL (bed volume) of mannose-*C*-glycoside resin was placed in a stoppered 1 mL spin column and equilibrated by washing with 0.5 mL lectin buffer three times.
B2 The resin was incubated in the dark for 10 min at ambient temperature with 200 μL of lectin buffer containing 10 μg fluorescein-conjugated lectin.
B3 The column was un-stoppered and unbound lectin was allowed to wash out via gravity, generally over 2–5 min.
B4 This flow-through was collected along with three 200 μL washes with lectin buffer and three 200 μL elutions with lectin buffer containing 200 mM mannose.
• The fluorescent intensity of each sample was measured in a microplate via FLx800 Fluorescence Microplate Reader (BioTek) to compare retention and elution of labeled lectins.
B5 Fluorescence signal in elution samples that is in excess of the last washes is indicative of binding by the lectin and its subsequent release.

### Part 3: Bacterial glycan-affinity chromatography

This *C*-glycoside resin allows for isolation of bacterial glycan affinity from otherwise complex mixtures while quantifying and manipulating interactions with specific target glycans. This has broad application for assessing glycan affinity, specificity, and mechanisms of displacement or competition. As numerous studies of glycan-mediated detection methods focus on the well-characterized interaction of *Escherichia coli* type-I fimbriae with mannosides, we have validated our platform with immobilized alpha-D-mannose and a non-pathogenic laboratory strain of *E. coli.*

**Fig. 2:**
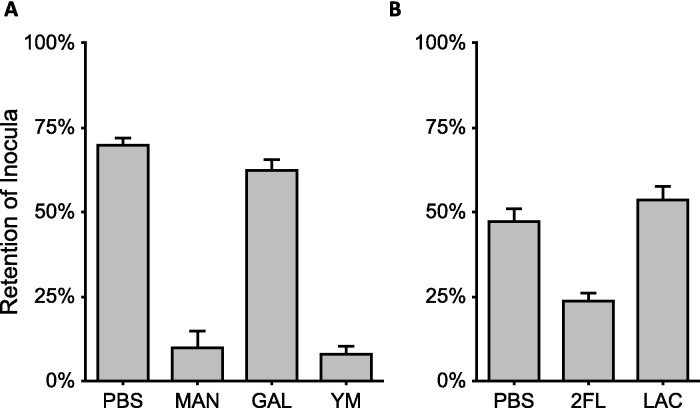
Retention of *E. coli* K12 on mannose affinity chromatography resin in the presence of soluble competitors, standardized as a fraction of the inoculum. (A) *E. coli* K12 adhesion to mannose-*C*-glycoside resin was inhibited only in the presence of soluble competitive mannosyl structures. (B) *Campylobacter jejuni* 11168 adhesion to 2FL-*C*-glycoside resin was inhibited only by the presence of soluble 2FL. PBS: phosphate buffered saline non-competitive control, MAN: mannose (50 mM) competition, GAL: galactose (50 mM) competition, YM: yeast mannan (20 mg/mL) competition, 2FL: 2′fucosyllactose (5 mg/mL) competition, LAC: lactose (5 mg/mL) competition. Data shown are averages ± standard error, experiments performed in triplicate.

Fig. 2A demonstrates the retention of *E. coli* K12 to mannose resin that is inhibited only by mannose-containing competitors. Bound cells could also be recovered for enumeration by elution with competing glycans, by altered pH, or denaturants, but we have found that the discrepancy between cell load of the inocula and the flow-through is more consistent and is sufficient for an accurate picture of initial retention.

Since the resin can be as easily constructed with any reducing carbohydrate, and no further modification of samples may be required, there is considerable potential for exploring alternative affinities of known and unknown organisms. To this end, we have also established that the resin is compatible with adhesion by less tractable organisms than *E. coli* to more complex glycan structures. Fig. 2B demonstrates the retention of *Campylobacter jejuni* NCTC 11168 to immobilized 2′fucosyllactose (2FL) in a carbohydrate specific manner, a specificity that has been demonstrated to a lesser extent on Caco-2 cell culture [6].

The same principles of glycan adhesion specificity can be applied as a selective screening tool for separating cells with differing affinity. A selective pressure for the adhesive affinity to target glycans could be beneficial to the exploration competitive exclusion or the directed evolution of high-affinity strains. The example we have used to demonstrate this feature reflects the phase-variation of the *E. coli* fimbrial operon. Cells that are fimbriated result in a colony morphology differentiable from those that are non-fimbriated [7], which can be verified by PCR amplification and digestion of the *fim* switch [8].

**Fig. 3:**
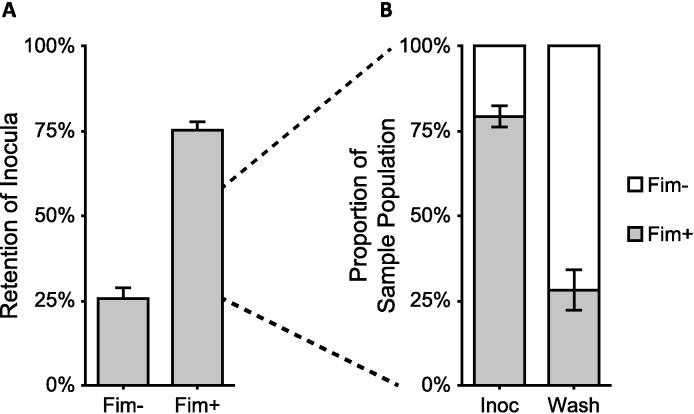
Retention of *E. coli* K12 on mannose affinity chromatography resin differs based on fimbriation of the inocula. (A) Inocula composed of fimbriated cells (Fim +) was recovered at a significantly lower level than non-fimbriated cells (Fim −), indicating they had a greater affinity for the resin. (B) Of the cells from a predominantly fimbriated inoculum (Inoc), the initial wash-through (Wash) shows a distinct enrichment of non-fimbriated cells. This demonstrates the resin's potential for separating populations based on selection for glycan adhesion. Data shown are averages ± standard error, experiments performed in triplicate.

Fig. 3 demonstrates the selective pressure of immobilized C-mannoside resin for the separation of *E. coli* K12 mixed phenotypes. On average, approximately 94–96% of fimbriated cells in a given population could be retained on the resin, while only 15–20% of non-fimbriated cells would remain, after the initial flow-through alone (data not shown). Phase-variability of the populations derived from fimbriated and non-fimbriated colonies may account for the deviation from these numbers that is seen in Fig. 3A, as even inocula prepared from fimbriated colonies were partially non-fimbriated (Fig. 3B).

#### Materials

• Mannose-*C*-glycoside and 2FL-*C*-glycoside ketohydrazide resins, as prepared above in Part 1
• Stoppered spin columns with 30 μm pore filters (Thermo Scientific cat. 69725)
• *E. coli* ATCC 29425 (K12)
• *C. jejuni* NCTC 11168 (Cj11168)
• Brain-Heart Infusion broth and agar plates (BHI; BD cat. 237500)
• Mueller Hinton broth (BD cat. 275710)
• Citrated bovine blood (Quad Five cat. 930)
• Phosphate buffered saline (PBS)
• Mannose
• Galactose
• Mannan from *Saccharomyces cerevisiae* (Sigma cat. M7504)
• Lactose
• 2′Fucosyllactose

#### Procedure – determining specificity of microbial adhesion

A1 Prepare bacterial cultures grown to mid-late exponential growth phase.
  a. *Escherichia coli* K12 was grown aerobically in BHI at 37 °C with 200 RPM shaking to an optical density at 600 nm (OD_600_) of 0.8–1.0.
  b. *C. jejuni* 11168 was grown under microaerophilic conditions as a lawn on Mueller Hinton-based blood agar plates with 5% bovine blood (MHB) at 37 °C for 24 h.
A2 Prepare columns as before for lectin affinity verification, instead washing three times with 0.5 mL PBS to equilibrate prior to each test.
a. Optional: non-specific binding can be pre-blocked with the addition of protein such as bovine serum albumin, but we have found this to be unnecessary for our bacterial samples.
A3 Harvest bacterial cells to prepare dilutions for application to the column.
  a. K12 culture was centrifuged at 3000 × *g* for 3 min then re-suspended in PBS to wash, repeating twice.
  b. Cj11168 culture was removed from MHB by a sterile inoculating loop and suspended in PBS with brief vortexing. Culture was centrifuged at 3000 × *g* for 5 min then re-suspended in PBS to wash, repeating twice.
A4 Prepare the inocula for the columns by diluting the washed bacterial cultures in PBS to obtain approximately 2 × 10^5^ CFU/mL.
  a. K12 and Cj11168 were standardized to an OD_600_ of 0.50 ± 0.01, resulting in approximately 4 × 10^8^ CFU/mL, which was then diluted accordingly.
A5 Immediately prior to application to the columns, combine 0.5 mL aliquots of inocula with test competitors of interest.
  a. K12 inocula were combined with either 100 mM mannose, 100 mM galactose, 40 mg/mL yeast mannan, or PBS as a control.
  b. Cj11168 inocula were combined with 10 mg/mL 2FL, 10 mg/mL lactose, or PBS as a control.
A6 Add the inocula to the columns and allow incubation until the resin settles, approximately 3 min.
A7 Open the columns and allow inocula to wash out via gravity until run dry, generally about 5 min.
A8 Collect the flow-through, plate on appropriate agar medium for overnight incubation, and enumerate to determine the amount of cells that were not retained in the column.
A9 Plate the inocula for enumeration on appropriate agar medium in parallel.
A10 Calculate bacterial retention as the fraction of the cells from the inoculum not recovered in the flow-through.

#### Procedure – screening microbial populations by glycan affinity

B1 *Escherichia coli* K12 was grown aerobically overnight on BHI agar at 37 °C.
B2 Colonies of fimbriated and non-fimbriated K12 were re-suspended in BHI broth to approximately OD_600_ 0.3.
B3 Culture was incubated until grown past OD_600_ 0.50, and then washed in PBS and standardized as above.
B4 Columns and bacterial dilutions were prepared as above and affinity assay was performed as before, but without combination of competing sugars.
B5 Enumeration of inocula and assayed samples included counting the colonies of fimbriated and non-fimbriated cells separately to assess their proportions.

*Note*: We have found that *C*-glycoside resin can be reused in multiple successive bacterial adhesion assays with no detectable decrease in effectiveness following washes with free competing ligand, detergent, heat, or other protein denaturants.

## Additional information

A number of tools have been demonstrated for investigating mechanisms of microbial glycan adhesion, including tissue culture [6,9], glycan microarrays [10,11], and other forms of chromatography [12,13]. As discovery platforms, however, many current techniques for exploiting glycan-mediated adhesion are not suitable for certain avenues of investigation. Microarrays require very low quantities of target ligand, but frequently have difficulty elaborating whole-cell interactions. For example, of whole-cell bacterial samples applied to glycan arrays from the Consortium for Functional Glycomics [14] the majority of results are inconclusive. Many arrays instead rely on the use of cell extracts and purified proteins. This often requires prior knowledge of expected interactions, including specific adhesive proteins or known adherent organisms and the optimal conditions for their growth and manipulation. Even when whole-cell adhesion is achievable, many methods do not facilitate further manipulation of the organisms. Such formats are not easily scalable or practical for enrichment in situ.

Models of *in vitro* tissue culture intent on elaborating glycan-mediated bacterial adhesion reflect *in vivo* conditions more closely, but cannot generally account for the presence of numerous other variables inherent to the cellular milieu and do not isolate specific glycan moieties that may be of interest [15]. Additionally, many tools require manipulation of organisms prior to use for proper detection, including most array and glycoparticle models. Alterations such as fluorescent staining or radiolabeling may have undetected consequences regarding adhesion or viability, but these effects are often ignored. Furthermore, many of these techniques also rely on complex, difficult, or hazardous chemical reactions for derivatization and immobilization of glycans that may not guarantee proper stereospecificity.

*C*-glycoside affinity resin is simple to construct and use, versatile, and sidesteps numerous pitfalls prevalent in methods of similar approach to glycan-bacteria adhesion. Optimization of the model for the affinity-based selection of probiotic organisms and the investigation of multiple glycan-pathogen interactions is currently underway, which is expected to facilitate the discovery and application of probiotic mechanisms for the prevention of pathogenic adhesion.
